# Association of Activity and Chronicity Indices With Clinical Presentation and Biochemical Parameters in Pediatric Lupus Nephritis

**DOI:** 10.7759/cureus.97237

**Published:** 2025-11-19

**Authors:** Nafisa Sermin, Mohammad Mosiur Rahman, Sultana Gulshana Banu, Raisa Badhan

**Affiliations:** 1 Clinical Pathology, Dhaka Medical College Hospital, Dhaka, BGD; 2 Pathology, Bangladesh Medical University, Dhaka, BGD; 3 Microbiology, National Institute of Burn and Plastic Surgery, Dhaka, BGD

**Keywords:** activity index, autoimmune disease, chronicity index, lupus nephritis, systemic lupus erythematosus

## Abstract

Background

Systemic lupus erythematosus is an autoimmune inflammatory multisystem disorder, with the clinical presentation of childhood lupus nephritis varying widely. The underlying histological severity of pediatric lupus nephritis cannot always be predicted.

Methodology

This cross-sectional, observational study was conducted at the Department of Pathology, Bangladesh Medical University (BMU). The study included clinically diagnosed cases of pediatric lupus nephritis from March 2021 to January 2023. Patients were enrolled by consecutive sampling after fulfilling the inclusion and exclusion criteria. Light microscopy and immunofluorescence findings of the formalin-fixed paraffin-embedded renal biopsy specimens were evaluated and recorded. The statistical analysis was performed using SPSS version 22.0 for Windows (IBM Corp., Armonk, NY, USA).

Results

Among 80 patients with lupus nephritis, the majority (67.5%) belonged to the age group 13 to 18 years. The mean age was 13.96 ± 3.09 years, ranging from 6 to 18 years. Overall, 83.75% of patients were female, and 16.25% were male. Active lesions were more frequently seen (60%) than chronic lesions (12.5%). The majority (37.5%) of the patients presenting with isolated proteinuria showed the presence of activity indices. Patients with nephritic-nephrotic syndrome and isolated proteinuria (40% each) showed the presence of chronicity indices. However, no clinical features were significantly associated with activity and chronicity indices. Among the biochemical parameters, 24-hour urinary total protein was found to be significantly associated with the activity index.

Conclusions

In this study, a significant proportion of patients were female and in the adolescent age group. Urinary total protein was found to be significantly associated with the activity index. Four of the activity indices and one of the chronicity indices were found to be statistically significant.

## Introduction

Onset of systemic lupus erythematosus (SLE) during childhood and adolescence comprises about 15-20% of all SLE cases [1]. Disease severity and multiorgan manifestations are more pronounced in patients in this age group than in the adult patient group [2]. In the pediatric age group, the clinical presentation of lupus nephritis is wide, ranging from asymptomatic hematuria, mild proteinuria, nephrotic or nephritic syndrome, rapidly progressive glomerulonephritis, acute and chronic renal failure, to end-stage renal disease [3]. Among the serological markers, antinuclear antibodies (ANAs) are found to be positive in more than 95% of patients and are most characteristic of SLE diagnosis [4]. However, the clinical and serologic assessment cannot always predict the underlying histological severity. Renal biopsy, along with immunofluorescence study, can confirm the diagnosis of lupus nephritis, identify various histological patterns, provide prognostic information, and aid in formulating a treatment plan [5]. According to the Modification Proposal 2018 [6], six classes of lupus nephritis have been identified based on light microscopy and immunofluorescence findings in a renal biopsy sample having a minimum of 10 glomeruli. The modified National Institutes of Health (NIH) semi-quantitative scoring system of the activity and chronicity indices was introduced in this proposal. Activity and chronicity indices of the new modified semi-quantitative scoring system also serve as a prognostic tool and guide clinicians toward the treatment of lupus nephritis. For example, a high chronicity index shows therapeutic resistance and higher renal failure rates. On the other hand, lesions with a high activity index are more likely to respond to aggressive therapy and are potentially reversible [7]. Lupus nephritis is one of the major complications seen in patients with SLE, with a higher morbidity and mortality rate in the pediatric population. Early onset of lupus nephritis has become a matter of great concern in recent years. The adolescent population is at risk of developing a more severe form of lupus nephritis. In 2018, to improve therapeutic efficacy, the International Society of Nephrology/Renal Pathology Society (ISN/RPS) modified the 2003 classification system of lupus nephritis. This modified classification system eliminates the A, A/C, and C subdivisions of class III and IV, as these have no significant difference in terms of renal outcome [8]. The present study aimed to correlate the clinical and biochemical findings with activity and chronicity indices in patients up to 18 years of age.

## Materials and methods

Study design and setting

This cross-sectional, observational study was conducted in the Department of Pathology, Bangladesh Medical University (BMU), Dhaka, from March 2021 to January 2023. The study included prospective data of clinically diagnosed cases of pediatric lupus nephritis over the study period.

Sampling and study population

A consecutive sampling method was applied. The study population consisted of clinically and biochemically diagnosed cases of pediatric lupus nephritis aged up to 18 years. Only renal biopsy specimens containing at least 10 glomeruli on light microscopy examination were included to ensure adequate histopathological evaluation. Cases that had already received long-term immunosuppressive therapy for more than six months before sample collection were excluded from the study.

Study sample

A total of 80 cases were included. Paraffin blocks, histological slides, and relevant clinical information of the selected cases were collected from the Department of Pathology, BMU, and the Kidney Foundation Hospital and Research Institute, Dhaka.

Data collection technique

Two samples were received in the laboratory from each patient. One sample was preserved in 10% formalin for light microscopy. The other sample was kept in normal saline for an immunofluorescence study and was stored in a freezer; subsequent cryostat sectioning was done. All steps of the study and the collected data of the patients were saved properly by using appropriate measures and maintaining confidentiality.

Study procedure

Routine Processing and Staining for Light Microscopy

Tissue in formalin was embedded in liquid paraffin and processed routinely. Subsequently, sections were cut thin (3-5 µm) with a microtome and stained with hematoxylin and eosin (H&E), periodic acid-Schiff (PAS), Masson’s trichrome stain, and methanamine silver stain.

Immunofluorescence Staining and Microscopy

Tissue stored in the freezer was transferred to the cryostat cabinet, placed on a holder, embedded in an optimum cutting temperature compound, and quickly frozen at -20°C. Sections were cut in a -20°C cooled chamber at a thickness of 4-5 µm and picked up onto specially designed ring slides. The sections were air-dried and stained with properly diluted FITC-conjugated rabbit polyclonal anti-human IgG (catalog number: F0202), IgA (catalog number: F0204), IgM (catalog number: F0203), C3 (catalog number: F0201), and C1q (catalog number: F0254): (Dako, Glostrup, Denmark). Proper washing was done before and after each staining procedure. The slides were examined under a fluorescence microscope. The site and pattern of antibody deposition were recorded. The degree of fluorescence (intensity) was graded semi-quantitatively as trace, (+), (++), (+++), and (++++).

Histopathological Evaluation of Renal Biopsy Sections

Routine H&E, PAS, Masson’s trichrome, and silver-stained sections of the renal biopsy sample were examined. Sections were examined for changes in the following four components: glomeruli, tubules, interstitium, and blood vessels. The glomeruli were checked for cellularity, mesangium, basement membrane changes, segmental or global sclerosis, crescents, inflammatory cells, karyorrhectic debris, fibrinoid necrosis, hyaline thrombi, adhesion to Bowman’s capsule, and deposits. Epithelial changes and the presence of various types of casts in tubules, interstitial inflammation, fibrosis, tubular atrophy, and changes in the blood vessels were also noted. The histological diagnosis of each class was made according to the modified ISN/RPS 2018 classification system. Activity and chronicity indices were recorded from histological slides to grade the severity of individual histopathologic features on a 0 to 3+ scale. Histological and immunofluorescence findings were correlated with available clinical findings and laboratory investigation reports before making a final diagnosis.

Data recording

All patient data were recorded methodically in a data sheet. A separate data collection sheet was used for each patient.

Statistical analysis

After collection, all the required data were checked, verified for consistency, and tabulated using SPSS version 22.0 for Windows (IBM Corp., Armonk, NY, USA). Descriptive statistics (frequency and percentages) were used to summarize the patients’ demographic and clinical characteristics and are presented as tables and figures. Statistical significance was considered at a 95% confidence level. Continuous variables were expressed as mean with standard deviation and range. In addition, categorical variables were expressed as frequencies and percentages. To test any association, the chi-square test or the unpaired t-test was used. In all cases, the significance level was considered at a p-value <0.05.

Ethical considerations

Before starting this study, the research protocol was approved by the Institutional Review Board of BMU (former Bangabandhu Sheikh Mujib Medical University), Dhaka (approval number: BMU/2021/12786, date: 19.12.2021). Precautions were taken to protect the confidentiality of the participants. Informed written consent was obtained from the parents of the patients without any influence.

## Results

Among 80 patients with lupus nephritis, the majority (67.5%) belonged to the adolescent age group (13- 18 years). The mean age was 13.96 ± 3.09 years, ranging from 6 to 18 years. More than four-fifths (83.75%) of the patients were female, and 13 (16.25%) patients were male (Table 1).

**Table 1:** Distribution of the study patients by demographic profile (n = 80).

Variables	Frequency (n)	Percentage (%)
Age (years)		
Childhood (1–12 years)	26	32.5
Adolescence/Teenage (13–18 years)	54	67.5
Mean ± SD	13.96 ± 3.09	-
Minimum–maximum	6–18	-
Gender		
Male	13	16.25
Female	67	83.75

**Figure 1:**
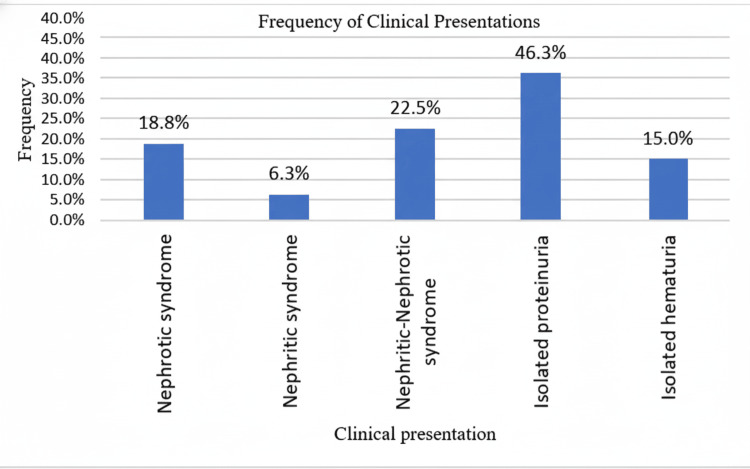
Bar chart showing the frequency of different clinical presentations of lupus nephritis.

The majority (46.3%) of lupus nephritis patients presented with isolated proteinuria, 22.5% with nephritic-nephrotic syndrome, 18.8% with nephrotic syndrome, 15% with isolated hematuria, and a minority (6.3%) with nephritic syndrome (Figure 1).

The urinary 24-hour total protein ranged between 0.10 and 5.7 g/day. Similarly, serum creatinine had a range between 0.37 and 3.39 mg/dL in our study group (Table 2).

**Table 2:** Biochemical parameters in the study patients (24-hour urinary total protein and serum creatinine levels) (n = 80).

Parameters	Mean ± SD	Minimum–maximum
24-hour urinary total protein (g/day)	2.24 ± 1.44	0.10–5.70
Serum creatinine (mg/dL)	0.90 ± 0.47	0.37–3.39

Low serum C3 was found in 86.3% cases, and low serum C4 was found in 86.3% cases. Anti-dsDNA and ANA were positive in all cases (Table 3).

**Table 3:** Biochemical parameters in the study patients (antibody and complement levels) (n = 80).

Parameters	Frequency (n = 80)	Percentage (%)
Anti-dsDNA	80	100.0%
Antinuclear antibodies	80	100.0%
Serum C3 (low)	69	86.3%
Serum C4 (low)	69	86.3%

All of the 80 cases showed positive fluorescence findings. IgG was the most frequent deposit (95%), with the majority of 3+ intensity (55.0%). Other positive findings were C1q (71.3%) with a majority (37.5%) of 2+ deposit, C3 (63.8%) with a majority (35%) of 1+ deposit, IgA (62.6%) with a majority (35%) of 2+ deposit, and IgM (48.8%) with a majority (30.0%) of 1+ deposit (Table 4).

**Table 4:** Frequency distribution of different immune deposits on direct immunofluorescence (DIF) study (n = 80).

DIF deposit	Frequency (n)	Percentage (%)	Total deposit (%)
IgG			
1+	4	5.0	95
2+	25	31.3	
3+	44	55.0	
4+	3	3.8	
IgM			
Trace	14	17.5	48.8
1+	24	30.0	
2+	14	17.5	
3+	1	1.3	
IgA			
Trace	11	13.8	62.6
1+	21	26.3	
2+	28	35.0	
3+	1	1.3	
C3			
Trace	9	11.3	63.8
1+	28	35.0	
2+	19	23.8	
3+	4	5.0	
C1q			
Trace	1	1.3	71.3
1+	11	13.8	
2+	30	37.5	
3+	16	20.0	

In this study, the highest total value of activity index was observed in class IV lupus nephritis, and the value was 13. Among 22 cases of class IV lupus nephritis, the activity index had a wide range of values (4-13). The highest total value of chronicity indices was found in class III lupus nephritis (4). No active or chronic lesions were present in class I lupus nephritis (Table 5).

**Table 5:** Distribution of activity and chronicity indices in the study group (n = 80).

Histological classes	Activity index	Chronicity index
	Mean ± SD (range)	Mean ± SD (range)
I	0.00 ± 0.00 (0.00–0.00)	0.00 ± 0.00 (0.00–0.00)
II	1.81 ± 1.60 (0.00–6.00)	0.23 ± 0.43 (0.00–1.00)
III	4.71 ± 1.73 (2.00–8.00)	1.00 ± 1.24 (0.00–4.00)
IV	8.00 ± 2.49 (4.00–13.00)	1.23 ± 0.97 (0.00–3.00)
V	1.33 ± 0.78 (0.00–3.00)	0.50 ± 0.90 (0.00–3.00)
III + V	4.50 ± 1.26 (3.00–6.00)	0.50 ± 0.58 (0.00–1.00)
IV + V	6.00 ± 0.0 (6.00–6.00)	1.00 ± 0.0 (1.00–1.00)

Among the histological features of the activity index, neutrophils/karyorrhexis was the most frequent, which was present in 62 (77.5%) cases, followed by interstitial inflammation in 59 (73.8%) cases and endocapillary hypercellularity in 48 (60.0%) cases. Among the histological features of chronicity index, the most common finding was interstitial fibrosis (30.0%), followed by tubular atrophy (25%) and total glomerulosclerosis (11.2%). Fibrous crescents were found in a minority (2.5%) of cases (Table 6).

**Table 6:** Distribution of different histological features of activity and chronicity indices in the study group (n = 80).

	Frequency (n)	Percentage (%)
Activity indices		
Endocapillary hypercellularity	48	60.0
Neutrophils/Karyorrhexis	62	77.5
Fibrinoid/Necrosis	6	7.5
Hyaline deposits	15	18.8
Cellular/Fibrocellular crescents	17	21.3
Interstitial/Inflammation	59	73.8
Chronicity indices		
Total glomerulosclerosis	9	11.2
Fibrous crescents	2	2.5
Tubular atrophy	20	25.0
Interstitial fibrosis	24	30.0

In this study, the majority (37.5%) of the patients presenting with isolated proteinuria showed the presence of activity indices. However, no clinical features were significantly associated with activity indices (Table 7).

**Table 7:** Association of activity index with clinical presentation. The chi-square test (χ² = 4.12, df = 4, p > 0.05) was done. s = significant (as p-value <0.05); ns = not significant (as p-value >0.05).

Clinical presentation	Activity index		P-value*
	Present (n = 48), n (%)	Absent (n = 32), n (%)	
Nephrotic syndrome	9 (18.8)	6 (18.8)	1.000 (ns)
Nephritic syndrome	4 (8.3)	1 (3.1)	0.643 (ns)
Nephritic-nephrotic syndrome	15 (31.3)	3 (9.4)	0.059 (ns)
Isolated proteinuria	18 (37.5)	19 (59.4)	0.056 (ns)
Isolated hematuria	7 (14.6)	5 (15.6)	0.898 (ns)

Patients with nephritic-nephrotic syndrome and isolated proteinuria (40% each) showed the presence of chronicity indices. However, no clinical features were significantly associated with chronicity indices (Table 8).

**Table 8:** Association of chronicity index with clinical presentation. The chi-square test (χ² = 3.27, df = 4, p > 0.05) was done. s = significant (as p-value <0.05); ns = not significant (as p-value >0.05).

Clinical presentation	Chronicity index		P-value*
	Present (n = 10), n (%)	Absent (n = 70), n (%)	
Nephrotic syndrome	2 (20.0)	13 (18.6)	0.914 (ns)
Nephritic syndrome	0 (0.0)	8 (11.4)	1.000 (ns)
Nephritic-nephrotic syndrome	4 (40.0)	14 (20.0)	0.220 (ns)
Isolated proteinuria	4 (40.0)	33 (47.1)	0.745 (ns)
Isolated hematuria	2 (20.0)	10 (14.3)	0.641 (ns)

Among the biochemical parameters, 24-hour urinary total protein was found to be significantly associated with activity index (p = 0.010) (Table 9).

**Table 9:** Association of activity index with biochemical parameters. ^a^: Unpaired t-test (t = 2.64, df = 78) and ^b^chi-square test (χ² = 0.74, df = 1) were done. s = significant (as p-value <0.05); ns = not significant (as p-value >0.05).

Biochemical parameters	Activity Index		P-value
	Present (n = 48)	Absent (n = 32)	
Urinary total protein (mean ± SD), g/day	2.61 ± 1.40	1.69 ± 1.36	0.010 (s)^a^
Serum creatinine (mean ± SD), mg/dL	0.98 ± 0.55	0.80 ± 0.31	0.090 (ns)^a^
Serum C3 (low), n (%)	41 (95.3)	28 (87.5)	0.392 (ns)^b^
Serum C4 (low), n (%)	41 (95.3)	28 (87.5)	0.392 (ns)^b^

No biochemical parameter other than this was significantly associated with activity or chronicity indices (Table 10).

**Table 10:** Association of chronicity index with biochemical parameters. ^a^: Unpaired t-test (t = 1.14, df = 78) and ^b^chi-square test (χ² = 1.00, df = 1) were done. s = significant (as p-value <0.05); ns = not significant (as p-value >0.05).

Biochemical parameters	Chronicity index		P-value
	Present (n = 10)	Absent (n = 70)	
Urinary total protein (mean ± SD), g/day	2.73 ± 1.42	2.16 ± 1.45	0.257 (ns)^a^
Serum creatinine (mean ± SD), mg/dL	0.91 ± 0.37	0.91 ± 0.49	a0.969 (ns)^a^
Serum C3 (low), n (%)	10 (100.0)	59 (90.8)	0.317 (ns)^b^
Serum C4 (low), n (%)	9 (100.0)	60 (92.3)	0.389 (ns)^b^

## Discussion

This cross-sectional study, conducted in the Department of Pathology, BMU (formerly BSMMU), Dhaka, Bangladesh, explored the clinicopathological spectrum, immunofluorescence characteristics, and histopathological indices of activity and chronicity in pediatric lupus nephritis. The majority of patients were adolescents (67.5%) and female (83.75%), highlighting a marked female preponderance even in the pediatric age group, consistent with findings from several regional and international studies [8,9]. This gender pattern may be attributed to hormonal and genetic factors influencing disease susceptibility, particularly estrogen-mediated immune responses that enhance autoantibody production. The predominance of adolescent onset is also in line with global epidemiological data indicating that pediatric lupus nephritis typically manifests during the second decade of life, when hormonal and immune factors intersect [9,10]. Histologically, class II lupus nephritis was the most common (32.5%), followed by class IV (27.5%), which is in accordance with some previous pediatric studies [11,12] but differs from others reporting class IV as the predominant subtype [7,9]. In contrast, adult studies have consistently shown class IV as the leading histological subtype [13], reflecting a more aggressive disease course in older patients. Notably, five cases showed overlapping histological patterns, mainly class III + V and IV + V, a finding similar to that of a previous study [14], suggesting possible disease evolution or transition between proliferative and membranous forms, which might require refinement of classification criteria in future updates.

Clinically, the most frequent presentation was isolated proteinuria (46.3%), particularly in patients with class II (51.9%) and class V (66.7%) lesions, whereas class IV cases most commonly presented with nephritic-nephrotic syndrome (42.9%). This pattern aligns with findings reported by other studies, though in adult populations, nephrotic syndrome is more dominant [13,15]. These differences may reflect the earlier stage of disease recognition in children and the influence of immune complex deposition patterns. The biochemical findings also provided valuable diagnostic insight. The mean 24-hour urinary total protein was 2.24 ± 1.44 g/day, highest in class III + V (3.21 ± 2.20 g/day), showing a statistically significant association with the activity index, whereas serum creatinine averaged 0.9 ± 0.47 mg/dl, being highest in class III + V and class IV cases, reflecting greater glomerular involvement [16]. These results underscore that quantitative proteinuria remains an excellent non-invasive marker for disease activity, correlating with active histopathological lesions. However, no clinical features were significantly associated with either activity or chronicity indices, suggesting that biochemical and histological assessments are more reliable indicators of renal disease severity than clinical presentation alone.

All cases were positive for ANA and anti-dsDNA antibodies, confirming their diagnostic utility in pediatric lupus nephritis, while reduced serum complement (C3 and C4) levels were observed in 86.3% of patients, comparable to previous findings [11]. Immunofluorescence analysis revealed that IgG (95%) was the most frequent immunoreactant with 3+ intensity, followed by C1q (71.3%), with a “full-house” pattern (IgG, IgA, IgM, C3, and C1q positivity) seen in 65% of cases, which is consistent with both pediatric and adult studies [8,9,17]. The presence of C1q and full-house deposition is a hallmark of immune complex-mediated glomerulonephritis, reflecting the polyclonal nature of immune activation in SLE. Interestingly, the degree of fluorescence intensity in C1q and IgG correlated with higher activity indices, reinforcing their role as indicators of disease activity and potential prognostic markers.

Regarding histopathological indices, the mean activity index was 4.71 ± 1.73 in class III and 8.0 ± 2.49 in class IV, while the mean chronicity index was 1.0 ± 1.24 in class IV, indicating that proliferative forms (especially class IV) exhibit more active inflammation than chronic scarring. This finding corresponds with those reported by Hashmi et al. [8], suggesting that class IV lupus nephritis typically demonstrates severe histopathological activity but remains amenable to aggressive immunosuppressive therapy if detected early. In this study, active lesions were more frequent (60%) than chronic lesions (12.5%), with neutrophils/karyorrhexis (77.5%), interstitial inflammation (73.8%), and endocapillary hypercellularity (60.0%) being the most common features, whereas chronic lesions included interstitial fibrosis (30%), tubular atrophy (25%), and glomerulosclerosis (11.2%). These observations are consistent with other studies [13,15,17], supporting that pediatric lupus nephritis tends to present with predominantly active lesions that have not yet progressed to chronic fibrosis. Importantly, significant associations were found between activity indices and histological classes, and between interstitial fibrosis and chronicity indices, confirming previous findings and emphasizing the prognostic value of these indices in predicting renal outcomes.

The comparison of ISN/RPS 2003 and 2018 classification systems indicates that the modified NIH activity and chronicity indices proposed in the 2018 revision may provide a more reproducible and sensitive evaluation of disease severity across all classes of lupus nephritis [6]. In particular, the inclusion of additional interstitial parameters and clearer scoring systems enhances prognostic accuracy and helps standardize biopsy reporting across centers. Taken together, the current findings highlight that pediatric lupus nephritis in Bangladesh is characterized by a high proportion of active lesions, frequent IgG and C1q deposition, and significant correlation between urinary protein excretion and histological activity. Early renal biopsy remains the cornerstone of accurate classification and management, allowing timely initiation of aggressive immunosuppressive therapy before irreversible damage develops. Therefore, a comprehensive evaluation incorporating both morphological indices and biochemical parameters is essential for improving long-term renal survival and overall prognosis in children with lupus nephritis.

In the present study, a significant positive correlation was found between 24-hour urinary total protein and the activity index, suggesting that higher levels of proteinuria are indicative of more active renal inflammation in pediatric lupus nephritis. Conversely, none of the evaluated clinical parameters exhibited a statistically significant association with either the activity or chronicity indices, implying that clinical manifestations alone may not accurately reflect the true histopathological severity of renal involvement in these patients.

The present study has some limitations. It was conducted on a limited population, which may restrict the generalizability of the findings to a wider demographic. Additionally, the absence of follow-up data limits the ability to assess long-term outcomes or changes over time. Future research should address these limitations by including larger, multicentric populations and incorporating genetic testing to enhance the accuracy and reliability of the results.

## Conclusions

In this study, a significant correlation was found between 24-hour urinary total protein and the activity index, suggesting that higher proteinuria reflects more active renal involvement in pediatric lupus nephritis. In contrast, none of the assessed clinical features demonstrated a statistically significant association with either the activity or chronicity indices, suggesting that clinical presentation alone may not reliably predict the underlying histopathological severity of renal lesions in these patients. Though 24-hour proteinuria is a useful non-invasive marker of histologic activity, biopsy with standardized activity index/chronicity index scoring remains essential for accurate risk stratification.
